# Modeling hospital infrastructure by optimizing quality, accessibility and efficiency via a mixed integer programming model

**DOI:** 10.1186/1472-6963-13-220

**Published:** 2013-06-16

**Authors:** David Ikkersheim, Marit Tanke, Gwendy van Schooten, Niels de Bresser, Hein Fleuren

**Affiliations:** 1 KPMG Plexus, Breukelen, The Netherlands; 2 FALW, VU University, Amsterdam, The Netherlands; 3 Tilburg University, Tilburg, The Netherlands

## Abstract

**Background:**

The majority of curative health care is organized in hospitals. As in most other countries, the current 94 hospital locations in the Netherlands offer almost all treatments, ranging from rather basic to very complex care. Recent studies show that concentration of care can lead to substantial quality improvements for complex conditions and that dispersion of care for chronic conditions may increase quality of care. In previous studies on allocation of hospital infrastructure, the allocation is usually only based on accessibility and/or efficiency of hospital care. In this paper, we explore the possibilities to include a quality function in the objective function, to give global directions to how the ‘optimal’ hospital infrastructure would be in the Dutch context.

**Methods:**

To create optimal societal value we have used a mathematical mixed integer programming (MIP) model that balances quality, efficiency and accessibility of care for 30 ICD-9 diagnosis groups. Typical aspects that are taken into account are the volume-outcome relationship, the maximum accepted travel times for diagnosis groups that may need emergency treatment and the minimum use of facilities.

**Results:**

The optimal number of hospital locations per diagnosis group varies from 12-14 locations for diagnosis groups which have a strong volume-outcome relationship, such as neoplasms, to 150 locations for chronic diagnosis groups such as diabetes and chronic obstructive pulmonary disease (COPD).

**Conclusions:**

In conclusion, our study shows a new approach for allocating hospital infrastructure over a country or certain region that includes quality of care in relation to volume per provider that can be used in various countries or regions. In addition, our model shows that within the Dutch context chronic care may be too concentrated and complex and/or acute care may be too dispersed. Our approach can relatively easily be adopted towards other countries or regions and is very suitable to perform a ‘what-if’ analysis.

## Background

In the Netherlands, just as in most other countries, the majority of curative health care is organized in hospitals. Currently, there are 94 hospital locations in the Netherlands, almost all of them offering the whole range of treatments, varying from placing ear tubes to cancer treatment and long-term support of patients with COPD.

Recent studies show that quality of care can be improved by concentration of care [1,2]. Within the concept of concentration, hospitals and physicians focus on the treatment of a selection of specific conditions, treating a substantial volume of patients annually. Thereby they achieve superior outcomes in terms of quality of care [1,2]. In the literature this so called ‘volume-outcome’ association is widely established; hospitals that treat a larger volume of patients annually achieve better outcomes for conditions ranging from AIDS to cholecystectomies [3]. Moreover, increased hospital specialization - meaning a hospital that only treats certain diagnosis such as cancer or cardiovascular diseases - is associated with improved patient outcomes after adjusting for hospital procedural volume [4,5].

Concentration and specialization may also increase the efficiency of hospitals. One could expect efficiency gains due to a higher quality of care as costly complications may be avoided in centers that have superior expertise [6]. Economies of scale within a certain (group of) condition(s) can lead to additional cost reductions, i.e. by better procurement rates and higher turnover rates of tools and capacity usage [7,8]. On the other hand a decrease in the number of providers may result in a lower level of competition possibly leading to higher prices of care [9].

In contrast to the gain in quality of care and efficiency, concentration of care may lead to decreased accessibility of care as the distance to the nearest hospital location may increase. Therefore gains in quality of concentration of care should outweigh the heightened travel costs for patients. Studies show that a significant part of patients are willing to travel distances for higher quality of care [10,11], a better patient experience [12] or shorter waiting times [13,14]. Nonetheless, studies also show that patient preferences differ and that some patients (up to 40%) are preferring hospital care closely to their home rather than a short waiting time further away, partly caused by previous satisfactory experiences in the nearest located hospital [13].

Considering that more complex conditions have a more profound volume-outcome association and that for other, mostly chronic diseases, care should be provided as close to home as possible. Therefore, the ‘ideal’ hospital landscape would accommodate the quality versus accessibility trade off with a different number of providers per (group of) condition(s). In addition, to ensure an efficient delivery of care these providers should be large enough to operate efficiently.

To create optimal society value in our health care system, one should therefore optimize three dimensions: quality, efficiency and accessibility of care [15]. In previous studies on allocation hospital infrastructure, the allocation is usually based on only one or two of these dimensions: accessibility and/or efficiency of hospital care [16-18]. In this paper, we explore the possibilities to include a quality function in the objective of a MIP model, to give global directions how the ‘optimal’ hospital infrastructure would be in the Dutch context. To our knowledge, this is the first attempt to include the recent literature regarding volume-outcome relationship together with accessibility and efficiency characteristics to model a new optimal hospital infrastructure from a societal perspective.

## Methods

Mathematical models are very suitable to make complex trade-offs. Location models are studied for a long time in science and industry. An excellent overview of the various forms of location models can be found in Revelle et al. and Melo et al.; they describe the use of location models in supply chain management [19,20]. In our study we use a mixed integer linear (MIP) programming approach in an AIMMS software environment with the CPLEX solver to solve the Dutch hospital infrastructure problem [21]. Objective, relations and constraints will be explained in the following sections.

Models are very suitable, not only to find the optimal allocation of infrastructure, but also to perform sensitivity and ‘what-if’ analysis. This should be kept in mind since this is an explorative study in which estimations of the volume-outcome relations based on expert opinions are used.

### Conditions and hospital locations

To create the ‘ideal’ hospital landscape from the trade-off between quality, accessibility and operational efficiency, we first identified the possible hospital locations in the Netherlands and conditions that should be allocated. In the new hospital landscape, 150 possible hospital locations were defined. This number was chosen taken into account that: 1) There are enough locations to provide health care close to home (the average travel time currently with 95 hospital locations is 14.9 minutes to the closest located hospital [22] 2) the number of possible locations would not become a serious constraint in the model and 3) the model does not become too complex. From the total of 150 locations, 88 of these locations are based on the 3-digit distinct zip code of the 95 current hospitals in the Netherlands. The other locations are chosen in such a way that the total amount of 150 locations are optimally distributed in the Netherlands. This means that the sum of the individual driving times for all inhabitants is as small as possible. The density of the population per zip code area was used to calculate the optimal location of the hospitals [23].

#### Allocated diagnosis groups based on ICD-9-CM codes

The total health care delivered in hospitals in the Netherlands was assigned to one of 30 diagnosis groups. These diagnosis groups are slightly modified from the diagnosis groups of the International Classification of Diseases, Ninth Revision, Clinical Modification (ICD-9-CM, WHO), in such a way that the groups are as homogeneous as possible with regard to:

•the volume-outcome relationship.

The diagnosis group ‘Signs, Symptoms, and Ill-defined Conditions’, was excluded because of a lack of information. The ICD-9-CM codes and corresponding 30 diagnosis groups are described in Table 1. We formulated one obligatory relationship between ICD-9 groups in the model: between gynecology and pediatric care (groups 21 and 23, see Table 1). In the current model, all other diagnosis groups can operate on their own or be combined with other diagnosis groups if the efficiency constraint is not met. Details of the criteria of the volume-outcome relationship, the use of resources, and the accessibility are described in the paragraphs below.

**Table 1:** ICD-9 classification and diagnosis groups used in the present study

**Main category ICD-9-CM classification**	**The 30 diagnosis groups**	
Infectious and Parasitic Diseases	1	Acute infectious and parasitic diseases and poisoning
	2	Chronic infectious and parasitic diseases (tuberculosis, HIV, hepatitis)
Neoplasms	3	Neoplasms
Endocrine, Nutritional, and Metabolic Diseases and Immunity Disorders	4	Diabetes
	5	Endocrine, metabolic and immunity disorders
Diseases of Blood and Bloodforming Organs	6	Haematology
Mental Disorders	7	Acute mental disorders (i.e. psychosis)
	8	Chronic mental disorders (i.e. rehabilitation programs, personality disorders)
Diseases of the Nervous System and Sense Organs	9	Diseases of the nervous system
	10	Eyecare
	11	Earcare
Diseases of the Circulatory System	12	Cerebral hemorrhage and ischemia
	13	Chronic cardiovascular disease (including congestive heart failure)
	14	Cardiovascular disease with intervention (PCI/CABG)
Diseases of the Respiratory System	15	Acute diseases of the respiratory system (i.e. pneumonia and influenza)
	16	Complex pulmonary surgery
	17	Common surgery of the respiratory system (i.e. tonsillitis)
	18	Chronic obstructive pulmonary disease and allied conditions
Diseases of the Digestive System	19	Surgery of diseases of the digestive system
	20	Diseases of oral cavity, salivary glands, and jaws
Diseases of the Genitourinary System	21	Gynaecology
	22	Urinary system
Complications of Pregnancy, Childbirth, and the Puerperium	23	Complications of pregnancy, childbirth, and the puerperium
Diseases of the Skin and Subcutaneous Tissue	24	Dermatology
Diseases of the Musculoskeletal and Connective Tissue	25	Rheumatism and arthropathies (non surgical)
	26	Diseases of the musculoskeletal system that require surgical treatment
Congenital Anomalies	27	Congenital disorders
Newborn (Perinatal)	28	Certain conditions originating in the perinatal period
Injury and Poisoning	29	Emergency care (distorsion, luxation, common fractures)
	30	Specialized trauma care (injuries of organs, complex fractures)

### Data: volume-outcome relationship

In this study we estimated per diagnosis group the volume-outcome relationship compared to the diagnosis group neoplasms. This diagnosis group was chosen as a reference group, because the volume-outcome relationship is well studied for the condition breast cancer in the diagnosis group neoplasms and the angle of inclination of outcomes expressed as Quality Adjusted Lifetime Years (QALY’s) per volume step has been established in previous studies [22,24-31]. The study shows, in short, that on average 0.5 QALY for an individual may be gained per breast cancer treatment, when breast cancer care is concentrated from the current 94 hospitals (that each treat 138 cases per year) to 15 specialized breast cancer centers (that each treat 866 cases per year) centers. With this concentration of care the current Dutch 5-year survival for breast cancer could improve from 85% to 90% according to international literature and best practices^a^[22,24-32].

Although numerous studies have documented a volume-outcome relationship, literature to quantify the strength of this relationship is not available for all diagnosis groups. It is presumed that the association depends on the level of complexity of the intervention and the level of co-operation between different specialties [31,33]. Therefore based on the complexity of the diagnosis groups we divided the diagnosis groups in four categories and (somewhat arbitrarily) gauged volume-outcome relationship in QALYs relative to the diagnosis group neoplasms: high (the same relationship as the group neoplasms), intermediate (50% of neoplasms), low (5% of neoplasms), or no volume-outcome relationship.

The categories high, intermediate and low are established using the following criteria:

•High: multi medical specialty treatment, low volume, and high risk of complication, according to previous reports e.g. [34] and current volume norms per condition of professionals associations [35].

•Intermediate: a high complication risk but a single medical specialty approach [34].

•Low: groups with a high volume and a low risk of complication [34].

Since there is consensus in the literature that it is desirable that healthcare for chronic conditions is within close reach, chronic care was not included in this classification. As it requires frequent visits for relative low complex care, often accompanied with lifestyle change, chronic care was labeled as having no volume-outcome relationship [34].

After estimating the volume-outcome association per diagnosis group we calculated the angle of inclination of quality of care (expressed in QALYs) per diagnosis group, by simulating a concentration of care per diagnosis group from the current 94 locations to a virtual single centre (for the model formulation see below).

### Data: accessibility

For acute diagnosis groups, the travel time is in the Netherlands by law maximized on 45 minutes. Therefore we also used this as the maximum travel time in the acute diagnosis groups [36]. The maximum travel time for other diagnosis groups were set at 120 minutes. This amount is derived from Discrete Choice Experiments in which patients state they are willing to travel up to 230 kilometers (approximately 2 hours by car) for better quality of care [37].

### Data: use of resources

The average length of stay in the hospital is based on the national medical registry [38]. For the average lengths of stay in an Intensive Care Unit and the average duration of an operation we used the KPMG Plexus benchmark analysis of 70 hospitals in the Netherlands [39]. Use of resources, such as length of stay in the hospital, ICU admittance and use of operating rooms (OR). The minimum utilizations were estimated using benchmarking results from Dutch hospitals. For an OR the minimum utilization was estimated as being 65% of the total time available for operations (48 weeks a year, 5 days a week, 8 hours a day). For an ICU the minimum was estimated as having 6 beds occupied during 365 days a year. For a ward it was estimated as 80% utilization of 12 beds during 365 days a year.

### Data: costs per diagnosis group

The total costs per diagnosis group are estimated by the National Institute for Public Health and the Environment Cost of Illness in 2005 [40]. The abovementioned characteristics per diagnosis group are summarized in Table 2.

**Table 2:** The characteristics of the 30 diagnosis groups

**Diagnosis groups**		**Category**	**Costs (mlj euro)**	**Number of admissions**	**Volume- outcome relation**	**Maximum travel time (min)**
*Source:*			*Poos* et al. [40]	*LMR registry*[38]	*expert opinion*	*expert opinion*
1	Acute infectious and parasitic diseases and poisoning	Acute	95,8	93193	Intermediate	45
2	Chronic infectious and parasitic diseases	Chronic	184,4	3885	High	120
3	Neoplasms	Chronic	1845,1	405124	High	120
4	Diabetes	Chronic	197,6	17445	Non existent	45
5	Endocrine, metabolic and immunity disorders	Chronic	220	46837	Intermediate	45
6	Haematology	Elective/Chr.	146,1	51064	High	45
7	Acute mental disorders	Acute	85,7	12000	High	45
8	Chronic mental disorders	Chronic	306,6	22134	Non existent	45
9	Diseases of the nervous system	Chronic	522,2	89335	Low	45
10	Eyecare	Elective	596,9	207327	Low	45
11	Earcare	Elective	230,4	61090	Intermediate	45
12	Cerebral hemorrhage/ischemia	Acute	436,3	47925	High	45
13	Chronic cardiovascular disease (including congestive heart failure)	Chronic	938,5	180186	Non existent	45
14	Cardiovascular disease with intervention (PCI/CABG)	Acute	1047,7	163275	High	45
15	Acute diseases of the respiratory system	Acute	246,7	64310	Low	45
16	Complex pulmonary surgery	Elective	305,6	20502	High	45
17	Common surgery of the respiratory system	Elective	122,1	74168	Low	45
18	Chronic obstructive pulmonary disease and allied conditions	Chronic	305,5	48236	Non existent	120
19	Surgery of diseases of the digestive system	Elective	422,7	215757	Intermediate	45
20	Diseases of oral cavity, salivary glands, and jaws	Chronic	1108,9	26366	Low	45
21	Gynaecology	Elective	481,6	82053	Low	45
22	Urinary system	Elective	497,1	151334	Low	45
23	Complications of pregnancy, childbirth and the puerperium	Elective	739,3	296513	Intermediate	45
24	Dermatology	Chronic	412,3	68625	Low	45
25	Rheumatism and arthropathies (non surgical)	Chronic	961,2	157265	Low	45
26	Diseases of the musculoskeletal system that require surgical treatment	Elective	857,3	238155	Intermediate	45
27	Congenital disorders	Chronic	216,1	25669	High	120
28	Conditions originating in the perinatal period	Elective	331,2	75980	High	120
29	Signs, symptoms and ill-defined conditions	-	2.746,30	346106	Intermediate	45
30	Emergency care	Acute	604,1	52605	Non existent	45
31	Specialized trauma care	Acute	475,6	77330	High	120

### The MIP model

The problem is formulated using a mixed integer linear programming model, whereby accessibility and quality are translated to euros. This will be further explained in this section. We solve the instances by using the CPLEX solver within the AIMMS development environment.

Objective function:

$$\max\;\sum_{d}\left(\mathrm{Qualit}y_{d}-\mathrm{Trave}l_{d}\right)$$

Constraints:

$$\begin{array}{c} \mathrm{Qualit}y_{d}=\mathrm{EQ}\;NP_{d\;}\left(a_{d}\;NL_{d}+b_{d}\right)\;\mathrm{for}\;\mathrm{all}\;d,\\ \mathrm{Trave}l_{d}=2\;\mathrm{ET}\;NV_{d}\sum_{p,q}NP_{p,d}^{D}\;Y_{p,q,d}\;TT_{p,q\;}\mathrm{for}\;\mathrm{all}\;d,\\ \;\mathrm{Xp}=1\\ \begin{array}{c} \sum_{p}Y_{p,q,d}\leq Z_{q,d}\;M\;\mathrm{for}\;\mathrm{all}\;q,d,\\ \sum_{d}Z_{q,d}\leq X_{q}\;M\;\mathrm{for}\;\mathrm{all}\;q,\\ \sum_{q}Z_{q,d}=NL_{d}\;\mathrm{for}\;\mathrm{all}\;d,\\ \begin{array}{c} Y_{p,q,d\;}TT_{p,q}\leq \max t_{d}\;\mathrm{for}\;\mathrm{all}\;p,q,d,\\ \sum_{p,d}NP_{p,d}^{D}\;Y_{p,q,d}\;U_{f,d}\geq \min u_{f}\;X_{p}\;\mathrm{for}\;\mathrm{all}\;f,p. \end{array} \end{array} \end{array}$$

See below in Table 3 for the description of the indices, parameters and decision variables.

**Table 3:** Description of indices, parameters & variables

**Notation**	**Description**
*Indices*	
*d*	Diagnosis group
*f*	Facility
*p*	Zip code area
*q*	Zip code area
*Parameters*	
*a*_*d*_	The slope of the (linear) volume-outcome relationship of diagnosis group *d*
*b*_*d*_	The constant term of the (linear) volume-outcome relationship of diagnosis group *d*
*EQ*	Euros per QALY
*ET*	Travel expenses per time unit
*M*	Big number *[1,000,000]*
*maxt*_*d*_	The maximum acceptable travel time for diagnosis group *d*
*minu*_*f*_	Minimum utilization per facility *f*
*NP*_*d*_	The number of patients per diagnosis group *d*
$NP_{p,d}^{D}$	The number of patients per diagnosis group *d* and zip code *p*
*NV*_*d*_	The total number of visits per patient having diagnosis *d*
*TT*_*p,q*_	The driving time from zip code *p* to zip code *q*
*U*_*fd*_	The amount of resources each patient within diagnosis group *d* makes use of facility *f*
*Dependent variables*	
*Quality*_*d*_	Total quality achieved for diagnosis group *d [euros]*
*Travel*_*d*_	Total travel costs made for diagnosis group *d [euros]*
*NL*_*d*_	The number of locations treating diagnosis group *d*
*Decision variables*	
*X*_*p*_	Whether a health care location is established in zip code *p*
*Z*_*q,d*_	Whether diagnosis group *d* is treated at potential hospital location with zip code *q*
*Y*_*p,q,d*_	Whether for diagnosis group *d* patients from zip code *p* are assigned to potential hospital location with zip code *q*

### Model formulation

The objective of our model is the trade-off between quality and accessibility of care (traveling time) in concentration and specialization of hospital care. This leads to the following objective function:

$$\max\sum_{d}\left(\mathrm{Qualit}y_{d}-\mathrm{Trave}l_{d}\right)$$

#### Quality: volume-outcome relationship

It is assumed that the volume-outcome relationships are linear functions (*ax + b*) depending on the number of locations (*NL*_*d*_) treating a diagnosis group *d* (*a*_*d*_*NL*_*d*_ *+ b*_*d*_). After solving the problem the optimal number of locations is compared with the interval and if necessary another interval (with another linear function) is chosen^a^.

QALY’s are converted into monetary units (euros), based on earlier described concepts, using € 50,000 per QALY (*EQ*) which is a relative low number as previous studies often use $100,000 per QALY [41]. To test the robustness of our results we also ran the model using € 20.000 per QALY and € 100.000 per QALY. Implicitly, this is also a sensitivity analysis for the assumptions we made for the volume-outcome relationship.

The volume-outcome relationship for a disease group *d* is now as follows:

$$\mathrm{Qualit}y_{d}=\mathrm{EQ}\;NP_{d}\;\left(a_{d}\;NL_{d}+b_{d}\right)\;\mathrm{for}\;\mathrm{all}\;d,$$

#### Accessibility

The second element in the objective is travel time. The travel time of patients is calculated based on the demographics per zip code [23], using similar approaches as previous studies measuring accessibility of care [42]. The Netherlands are divided in 794 zip code areas (the Dutch Frisian Islands are excluded). The percentage wise distribution of patients for every diagnosis group is assumed identical for these 794 zip code areas.

Total travel expenses per diagnosis group are based on the number of patients (*NP*^*D*^_*p,d*_ per diagnosis group *d* and zip code *p*), the total amount of time traveling (number of visits times travel time) and the travel expenses per time unit (*ET*).

The total number of visits per patient having diagnosis *d* (*NV*_*d*_) was taken from current guidelines in which the number of outpatient clinic visits (including possible radiotherapy/chemotherapy and follow-up visits). It is assumed that all treatments take place at one location, including follow-up visits. For the driving time to the hospital locations (*TT*_*p,q*_) the Dutch Drive Time Matrix was used [43].

The travel expenses per hour (*ET*) are derived from the costs of traveling by taxi (€2.20 per kilometer) [44] and the loss of income by traveling, using the national median income of €32,000 a year for 1,600 working hours [23]. We assumed that apart from the patient, a second person always accompanies the patient. Based on the literature regarding patient preferences – as mentioned in the introduction – we assumed that all patients are willing to travel as these travel costs are compensated by a higher quality of care. As we know that individual patient preferences regarding willingness to travel may differ, this is a limitation of our used data and approach.

For all diagnosis groups *d* a maximum is set – based on expert opinions – on the acceptable travel time (*maxt*_*d*_) for patients. The decision variable *Y*_*p,q,d*_, which assigns zip code areas to potential hospitals for a diagnosis group, is restricted to the domain *(p,q,d)* with:

$$Y_{p,q,d\;}TT_{p,q}\leq \max t_{d}\;\mathrm{for}\;\mathrm{all}\;p,q,d.$$

The costs for traveling for a diagnosis group *d* is as follows:

$$\mathrm{Trave}l_{d}=2\;\mathrm{ET}\;NV_{d}\;\sum_{p,q}NP_{p,d}^{D}\;Y_{p,q,d}\;TT_{p,q\;}\mathrm{for}\;\mathrm{all}\;d.$$

#### Constraint: efficient use of resources

Efficiency is regarded as operational efficiency concerning three facilities *f*:

•Operating room (OR)

•Ward

•Intensive care unit (ICU)

It is assumed that there are no capacity limitations for these facilities and that the same facility can be used for different diagnosis groups.

A healthcare location is considered efficient when a minimum utilization (*minu*_*f*_) is reached. How much a facility is used depends on the number of patients coming to this specific healthcare location *p* ($\sum_{p,d}NP_{p,d}^{D}Y_{p,q,d}$ where *NP*^*D*^_*p,d*_ is the number of patients with diagnosis *d* living in zipcode *q* and *Y*_*p,q,d*_ indicates to which healthcare location *p* these patients go for care) and how much each patient makes use of this facility (*U*_*f,d*_).

This leads to the following constraint:

$$\sum_{p,d}NP_{p,d}^{D}\;Y_{p,q,d}\;U_{f,d}\geq \min u_{f}\mathrm{Xp}\;\mathrm{for}\;\mathrm{all}\;f,p.$$

No ethical approval was required for this study.

## Results

**Figure 1:**
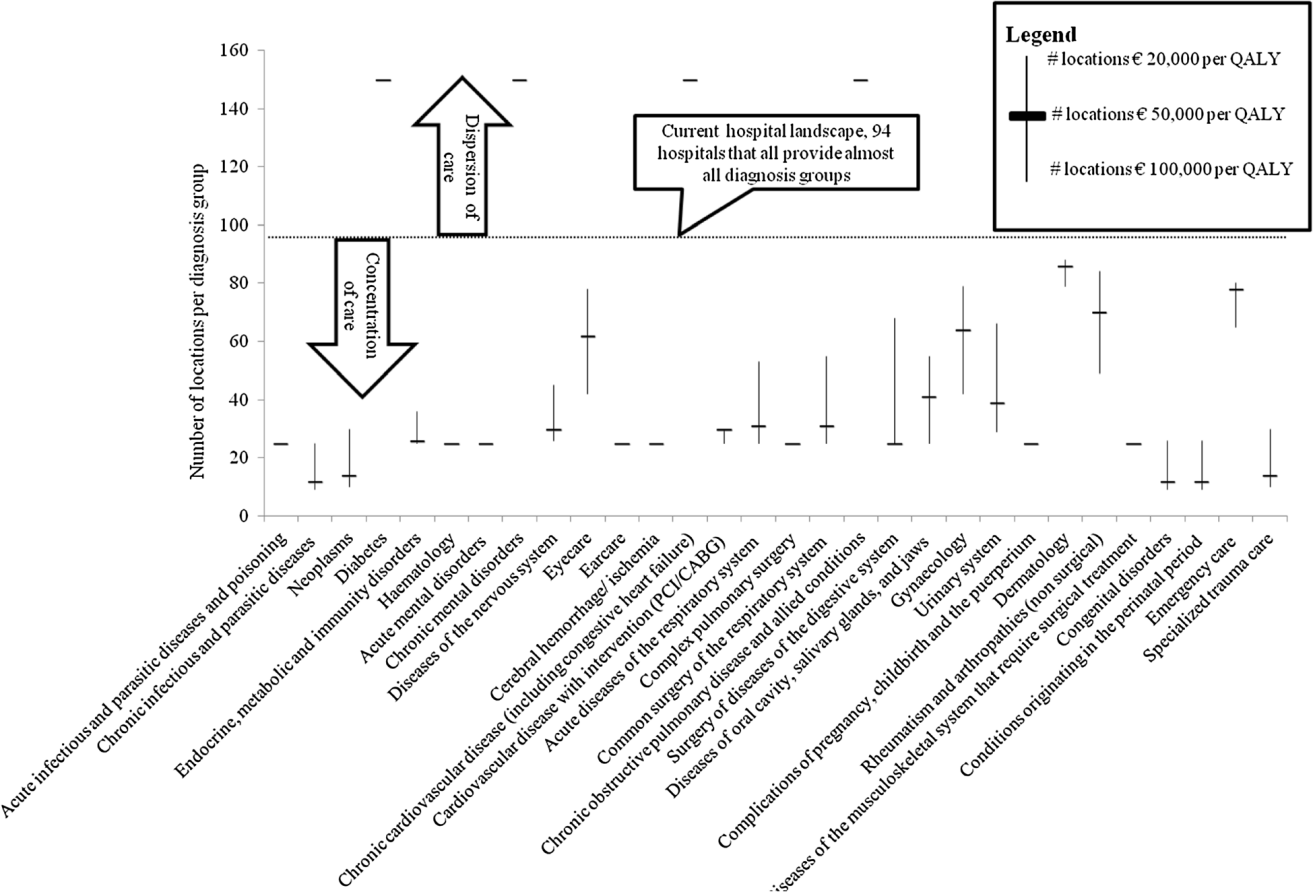
**Number of locations per diagnosis group, including sensitivity analyses with different values per QALY**^**1**^**. **^1^For some diagnosis groups the number of location did not alter with adjusting the monetary value per QALY to €P 20.000 and €100.000 (such as earcare). In such cases only the thick horizontal lines is displayed.

The above model has been run with the data as discussed. Figure 1 shows per diagnosis group the number of locations that ideally provides care for patients with a disease from that diagnosis group. The number of locations vary from 12 locations for diagnosis groups like congenital anomalies and 14 locations for neoplasms, which have a high volume-outcome relationship, to 150 locations for chronic diagnosis groups such as diabetes and chronic obstructive pulmonary disease (COPD). Also the figure displays the results from the model using € 20.000 per QALY and € 100.000 per QALY.

**Figure 2:**
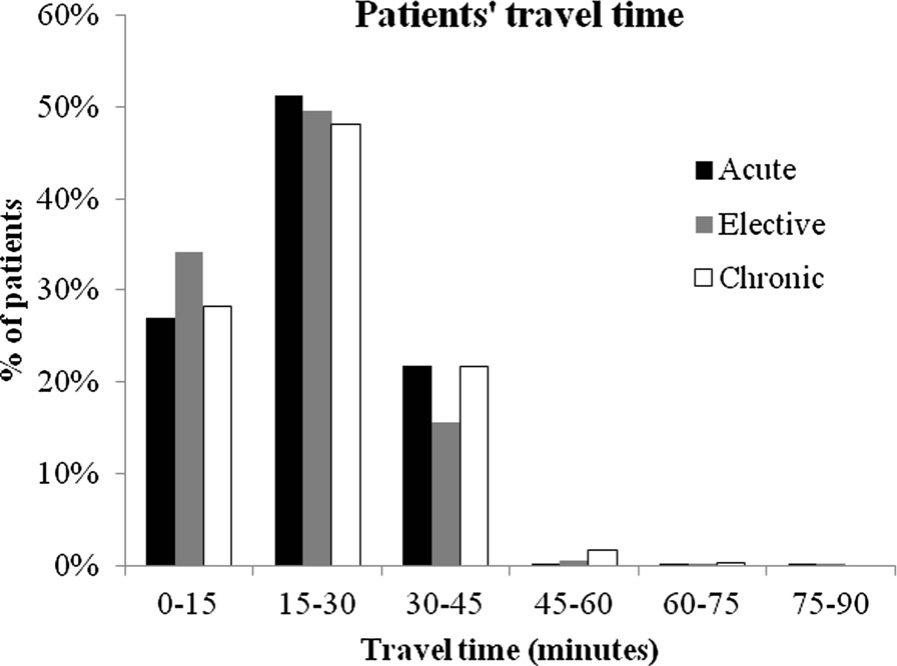
Travel time by car to nearest hospital per type of diagnosis group.

In the new hospital landscape, care for acute diagnosis groups and diagnosis groups with a strong volume-outcome relationship is provided on less locations than in the current situation. Patients with a chronic disease have less travel time than they have nowadays. Figure 2 shows the travel time for patients with acute, chronic or other diseases in the new hospital landscape. In the current landscape of 94 hospitals the average travel time is 14.9 minutes, whereas, given the number of locations per diagnosis group in Figure 1 with the scenario of € 50.000 per QALY the travel time will be 28.9 minutes.

## Discussion

In this study we explored how the optimal hospital landscape may look like based on a MIP model that tradeoffs the dimensions efficiency, quality and accessibility of hospital care in the Dutch context.

Our findings – given the assumptions we made - suggest that the current Dutch hospital landscape is too dispersed and too concentrated at the same time. For the more complex diagnosis groups such as neoplasms, patients would benefit from more concentration of care, where for chronic diagnosis groups such as COPD and diabetes optimal value may be created by making care more accessible to patients by providing chronic care at more locations throughout the country. These findings are in line with previous non empirical but rather conceptual studies regarding the Dutch hospital landscape that also advocates more dispersion for chronic care and more concentration for acute and/or complex diagnosis groups [45].

Based on our findings we believe that other countries or regions can apply our model as well.

Moreover we see that this development of concentration of care is actually happening in the Dutch hospital market; associations of medical specialists are formulating minimum volume norms for a range of conditions, resulting in fewer hospitals providing treatments for complex care [46]. In addition, there is a trend of merging of (smaller) hospitals, probably caused by efficiency pressures, concentration of care to enhance quality as well as market power considerations [37]. This latter development is not necessarily in line with the findings of our model as hospitals tend to merge as a whole, making no differentiation between characteristics of individual (groups of) diagnosis.

The main contribution of this study compared to previous hospital allocation studies is that it systemically includes the quality of care dimension [16-18]. Just these dimension is very highly rated by patients in multiple studies, that show that patients are willing to travel substantial distances for higher quality of care [11,47,48]. Therefore, including the quality of care dimensions in hospital allocation models that are used by policy makers, may improve the alignment of the hospital infrastructure with the preferences of society as a whole and individual patients.

It is important to state that this study has an explorative character especially because the volume-outcome relationship is not established yet for all different diagnosis groups let alone all the individual diagnosis. Future research regarding the volume-outcome relationship may alter the used input data and thereby alter the optimal number of locations per diagnosis group. In addition we did not include specialization effects of hospitals in our model and only ensured a minimal scale of hospitals but did not fully model economies of scale nor economies of scope effects. Also we did not include potential price effects due to a potentially lower level of competition between providers. However, most of these effects, like other constraints or functions, could be added to this approach as well: nearly the only aspect that needs to be taken into account is the size of the mathematical model (otherwise it cannot be solved within a reasonable time frame). Last, it is important to state that our model does not include individual preferences of patients, this implies that our findings of a different hospital landscape do not necessarily match with (all) individual preferences.

## Conclusion

In conclusion, our study shows a new approach for determining the allocation of diagnosis groups in a hospital blueprint in a country or certain region. This approach, considering quality of care in relation to volume per provider, can be used in various countries or regions. In addition, our model shows that within the Dutch context chronic care may be too concentrated while complex and acute care may be too dispersed.

## Endnotes

^a^ To calculate the gain in QALYs, three figures were used, information from national statistics and literature 1) A patient who survives 10 years after the diagnosis lives on for another 10 years on average. This is based on the average age of diagnosis in the Netherlands (60 years), and the average life expectancy at age 60 (about 23 years) The quality of life of breast cancer patients is between 80-90% when surviving 5 to 10 years, and 50% at most when dying within 5 to 10 years 3) The quality of life is equal for all patients that survived the diagnosis, regardless whether they are treated in high-volume or low-volume hospitals.

^b^ More accurate would be to regard the relationship as a continuous convex function. For modeling purposes a linear function is chosen that approaches the convex function in the interval of the expected number of optimal locations.

## Competing interests

The authors declare to have no competing interests.

## Authors’ contributions

DI drafted the manuscript and contributed to all other aspects of the study. MT, GvS, NdB and HF were involved in the data analyses and performed critical revision of the manuscript. All authors read and approved the final manuscript.

## Pre-publication history

The pre-publication history for this paper can be accessed here:

http://www.biomedcentral.com/1472-6963/13/220/prepub
